# Gut Microbiota and Their Metabolites in Acute Kidney Injury: Classification, Mechanisms, and Therapeutic Potential

**DOI:** 10.3390/metabo16090660

**Published:** 2026-09-09

**Authors:** Ziyi Qiu, Hao Zhang, Mengqing Ma, Binbin Pan, Changchun Cao

**Affiliations:** 1 Department of Nephrology, Sir Run Run Hospital, Nanjing Medical University, Nanjing 211100, China; qiuziyi@stu.njmu.edu.cn; 2 Department of Nephrology, Nanjing First Hospital, Nanjing Medical University, Nanjing 210006, China; zhanghao199208@njmu.edu.cn (H.Z.); mmq@njmu.edu.cn (M.M.); panbinbin@njmu.edu.cn (B.P.)

**Keywords:** acute kidney injury, gut microbiota, gut–kidney axis, metabolites, short-chain fatty acids, uremic toxins

## Abstract

Acute kidney injury (AKI) is a common critical syndrome with high morbidity and mortality, and a subset of patients may progress to chronic kidney disease. Recent studies have revealed that gut microbiota and their metabolites play pivotal roles in the pathogenesis of AKI. Under AKI conditions, the gut microbiota composition undergoes significant alterations, characterized by decreased beneficial bacteria and expansion of opportunistic pathogens, accompanied by impaired intestinal barrier and disordered microbial metabolism. Gut microbiota metabolites can be classified into protective metabolites (short-chain fatty acids, secondary bile acids, tryptophan metabolites, D-amino acids, and polyamines) and toxic metabolites (indoxyl sulfate, p-cresyl sulfate, trimethylamine N-oxide, and endotoxin). The former exert renoprotective effects through anti-inflammatory, antioxidant, and barrier-maintaining mechanisms, while the latter aggravate kidney injury via oxidative stress, inflammation activation, and hemodynamic disturbance. Based on the gut–kidney axis theory, interventions targeting gut microbiota (probiotics, prebiotics, fecal microbiota transplantation) and those targeting metabolites (supplementation of protective metabolites, removal of toxic metabolites) have shown promising prospects. This narrative review summarizes the characteristics of gut microbiota changes, classification and function of key metabolites, core mechanisms driving AKI, and microbiota-based intervention strategies, aiming to provide novel insights for early recognition and precision prevention of AKI.

## 1. Introduction

Acute kidney injury (AKI) is defined as a rapid decline in renal excretory function, manifesting as accumulation of serum creatinine and blood urea nitrogen or decreased urine output [1]. The etiology of AKI is diverse, involving prerenal factors (effective hypovolemia), renal factors (ischemia–reperfusion injury [IRI], nephrotoxic drugs), and postrenal factors (urinary tract obstruction). In low- and middle-income countries, infection and hypovolemic shock remain the main triggers; in high-income countries, AKI predominantly occurs in hospitalized elderly patients and is commonly associated with sepsis, drug exposure, or invasive procedures [2]. The incidence of AKI is approximately 10–15% among hospitalized patients and up to 30–60% in intensive care units [3]. Despite advances in therapeutic strategies, the morbidity and mortality of AKI remain high, and approximately 20–50% of patients progress to chronic kidney disease (CKD) or even end-stage renal disease (ESRD) [4,5]. Therefore, in-depth understanding of the pathogenesis of AKI and exploration of novel therapeutic strategies are of great significance.

Recent studies have revealed that gut microbiota and their metabolites play important roles in the occurrence and development of AKI. Dysbiosis can disrupt the intestinal barrier, facilitating the translocation of bacteria and their metabolites, such as lipopolysaccharide (LPS), into the bloodstream, thereby eliciting systemic inflammatory responses and aggravating kidney injury [4]. These microbiota changes are driven in part by uremic-toxin retention, altered intestinal pH and oxygen tension, reduced gut perfusion, and frequent antibiotic exposure in AKI, which collectively favor pathobiont overgrowth over commensal, fiber-fermenting taxa. Meanwhile, various microbiota-derived metabolites, including uremic toxins (trimethylamine N-oxide, indoxyl sulfate, p-cresyl sulfate), short-chain fatty acids, bile acids, and tryptophan derivatives, can directly or indirectly influence renal function by regulating inflammation, oxidative stress, cell death, and fibrosis. This narrative review summarizes the characteristics of gut microbiota changes, classification and function of key metabolites, core mechanisms driving AKI, and intervention strategies, aiming to reveal the complex network of the gut–kidney axis in AKI pathophysiology and provide novel insights for the prevention and treatment of AKI. Accordingly, the present work is a narrative rather than a systematic review: the literature discussed was identified through searches of PubMed, Web of Science, and Scopus using terms relating to acute kidney injury, gut microbiota, the gut–kidney axis, and microbial metabolites, and was selected on the basis of topical relevance rather than a pre-specified protocol.

## 2. Gut Microbiota Characteristics in Health and Acute Kidney Injury

### 2.1. Composition and Function of Gut Microbiota in Healthy State

Microorganisms are abundantly present in the human body, with the majority colonizing the intestine, where they play crucial roles in maintaining host homeostasis. The gut of healthy adults harbors a microbial community of up to one hundred trillion microorganisms, comprising nearly 1000 distinct species [5]. At the phylum level, the healthy gut microbiota is predominantly composed of Firmicutes, Bacteroidetes, Actinobacteria, and Proteobacteria, with Firmicutes and Bacteroidetes accounting for over 90% of the total microbiota [6]. The Firmicutes-to-Bacteroidetes (F/B) ratio is considered an important indicator of gut microbiota equilibrium, and healthy individuals typically maintain a relatively stable F/B ratio [7]. At the genus level, the healthy gut microbiota is enriched with beneficial genera, including *Bifidobacterium*, *Lactobacillus*, *Faecalibacterium*, *Roseburia*, and *Ruminococcus* [8].

In the healthy state, gut microbiota and the host maintain a dynamically balanced symbiotic relationship, with functions encompassing nutrient metabolism, immune regulation, and barrier defense. On one hand, gut microbiota participate in the fermentation of dietary fibers, producing short-chain fatty acids (SCFAs) and other beneficial metabolites that serve as energy sources for colonic epithelial cells and maintain host energy homeostasis by regulating enteroendocrine cells. On the other hand, gut microbiota participate in bile acid and tryptophan metabolism, producing metabolites that regulate host immune and inflammatory responses via specific receptor signaling pathways [9]. Furthermore, the biofilm formed by microbial colonization, together with the mucus layer and tight junction proteins, constitutes a triple mechanical–biological–chemical barrier that effectively defends against pathogen invasion. The stability of this microbiota–host symbiotic relationship is an important foundation for maintaining systemic immune homeostasis and organ function.

### 2.2. Gut Microbiota Changes in AKI

Upon AKI onset, the composition and abundance of gut microbiota undergo significant alterations, manifesting as decreased diversity, reduced beneficial bacteria, and proliferation of opportunistic pathogens. These shifts are driven by AKI-associated changes in the gut microenvironment itself—including retained uremic solutes, mucosal ischemia and edema, altered luminal pH and oxygen tension, and frequent antibiotic exposure—which together suppress obligate anaerobic, fiber-fermenting commensals while favoring facultative anaerobic pathobionts (metabolic consequences are detailed in Section 3). Based on 16S rRNA sequencing and metagenomic analysis, the alpha diversity index of AKI patients and animal models is significantly reduced [10]. At the phylum level, Firmicutes decrease while Bacteroidetes and Proteobacteria become enriched, with the F/B ratio markedly altered [11]. At the genus level, beneficial bacteria including *Bifidobacterium*, *Lactobacillus*, and *Faecalibacterium* decline, while opportunistic pathogens such as *Escherichia coli* and *Enterococcus* proliferate [12].

The microbiota changes in AKI exhibit temporal dynamics. Noel et al. demonstrated through longitudinal monitoring that gut microbiota undergo significant evolution at 72 h after IRI, with decreased relative abundance of Actinobacteria and dynamic changes in the Bacteroidetes-to-Firmicutes ratio, suggesting that dysbiosis worsens with AKI progression [13]. Yang et al. further found that the abundance of Proteobacteria in AKI patients positively correlated with serum creatinine levels and tubular injury scores, suggesting that specific microbiota changes may serve as potential biomarkers of AKI severity [14].

Different etiologies of AKI exhibit distinct microbiota characteristics. In ischemic AKI, the relative abundance of *Enterococcus* in the proximal small intestine can reach 60%, *Staphylococcus* in the distal small intestine accounts for approximately 50%, and *Akkermansia* is significantly enriched in the colon [15]. In sepsis-associated AKI, Enterobacteriaceae and Lachnospiraceae increase in abundance, and the elevated total microbial density positively correlates with AKI risk [16]. In cisplatin-induced AKI, SCFA-producing bacteria such as *Lactobacillus* and *Bifidobacterium* significantly decrease, while opportunistic pathogens such as *Escherichia–Shigella* increase [17]. These etiology-specific microbiota changes suggest that different AKI subtypes may require tailored microbiota modulation strategies.

Concomitant with structural changes, the metabolic function of gut microbiota is significantly remodeled. The reduction in SCFA-producing bacteria leads to insufficient production of protective metabolites such as butyrate and propionate; the enrichment of indole-producing and TMA-producing bacteria increases the generation of uremic toxins including indoxyl sulfate (IS), p-cresyl sulfate (PCS), and trimethylamine N-oxide (TMAO) [18]. Furthermore, intestinal wall edema, ischemia, and acid–base disturbances in AKI can further damage tight junctions, leading to downregulated expression of ZO-1 and occludin, increased intestinal permeability, and translocation of microbiota and their metabolites to renal tissue, directly participating in kidney injury [15]. This cascade of microbiota–metabolism–barrier disruption constitutes the pathophysiological basis of the gut–kidney axis in AKI.

## 3. Classification and Function of Gut Microbiota Metabolites in AKI

Gut microbiota produce a large number of bioactive small-molecule metabolites through the metabolism of dietary components and host-derived substances. Based on their effects on AKI progression, these metabolites can be broadly classified into protective and toxic categories (Table 1). The former mainly include SCFAs, secondary bile acids, tryptophan metabolites, D-amino acids, and polyamines, which exert renoprotective effects through anti-inflammatory, antioxidant, and barrier-maintaining mechanisms. The latter mainly include IS, PCS, TMAO, and LPS, which aggravate kidney injury via oxidative stress, inflammation activation, and hemodynamic disturbance. The imbalance between these two categories is a core component of gut–kidney axis disruption in AKI and represents a potential therapeutic target. This binary “protective” versus “toxic” framework is used here for clarity, but it is a simplification: several of these metabolites (e.g., tryptophan-derived indoles, D-amino acids) exert context-, dose-, tissue-, and disease-stage-dependent effects and can shift between beneficial and injurious roles, as noted for individual metabolites below.

### 3.1. Protective Metabolites

#### 3.1.1. Short-Chain Fatty Acids (SCFAs)

Short-chain fatty acids (SCFAs) mainly include acetate, propionate, and butyrate, produced by microbial fermentation of dietary fibers [14]. Under healthy conditions, the total SCFA concentration in the gut reaches 100–150 mmol/L, with acetate accounting for approximately 60% and propionate and butyrate each approximately 20%. The main SCFA-producing genera include *Bifidobacterium*, *Lactobacillus*, *Faecalibacterium*, *Roseburia*, and *Ruminococcus* [15]. In AKI, the abundance of these bacteria decreases, leading to reduced SCFA production. SCFAs are absorbed by intestinal epithelial cells and distributed via the portal vein to systemic organs including the kidney [16]. Propionate participates in gluconeogenesis, while acetate and butyrate participate in lipid synthesis and exert renoprotective effects [17].

Four SCFA receptors have been identified in the kidney: free fatty acid receptors GPR41 (FFAR3) and GPR43 (FFAR2), olfactory receptor Olfr78 (also known as 51E2), and niacin receptor GPR109a (HCA2). GPR41 and GPR43 are expressed in both renal tissue and renal arteries [48]; acetate can attenuate IRI-induced AKI by activating GPR43 [49]. GPR43 is also widely expressed on the surface of immune cells, including granulocytes, monocytes, dendritic cells, and mast cells, participating in immune regulation [50]. Olfr78 is mainly expressed in the juxtaglomerular apparatus and participates in renin secretion and blood pressure regulation [48]. GPR109a is a butyrate receptor whose activation promotes the differentiation of Treg cells and IL-10-producing T cells and inhibits the NF-κB pathway [19].

The renoprotective mechanisms of SCFAs involve multiple signaling pathways. In terms of anti-inflammatory effects, GPR43 activation inhibits the NLRP3 inflammasome and downregulates IL-1β and IL-18 expression [20]; as HDAC inhibitors, SCFAs promote Treg cell differentiation and inhibit the NF-κB pathway, alleviating renal inflammation [21]. Regarding barrier and energy metabolism, SCFAs upregulate tight junction proteins occludin and ZO-1, maintaining intestinal barrier integrity [51]; butyrate also upregulates MUC2 mucin expression via the HIF-2α signaling pathway [52], serves as the main energy source for colonic epithelial cells through β-oxidation to generate ATP [53], and activates the AMPK pathway to promote mitochondrial biogenesis [54]. In terms of hemodynamics, GPR41-mediated vasodilation and Olfr78-mediated renin secretion form a bidirectional regulation, maintaining renal hemodynamic stability [55]. Additionally, SCFAs promote the secretion of PYY and GLP-1 by enteroendocrine cells and regulate leptin secretion [20,56].

Multiple AKI animal models have confirmed the renoprotective effects of SCFAs. In the IRI model, acetate regulates dendritic cell and tubular cell function by activating GPR43 [12]; in cisplatin-induced AKI, butyrate improves mitochondrial membrane potential and attenuates tubular cell apoptosis [57]; in contrast-induced nephropathy, butyrate reduces IL-6 levels and blocks the NF-κB pathway [58]. Furthermore, in adriamycin nephropathy and anti-GBM glomerulonephritis models, butyrate also demonstrates significant renoprotective effects [59,60,61,62].

#### 3.1.2. Secondary Bile Acids

Primary bile acids are synthesized in the liver and converted to secondary bile acids by gut microbiota through deconjugation and 7α-dehydroxylation, mainly including lithocholic acid (LCA), deoxycholic acid (DCA), and ursodeoxycholic acid (UDCA) [59]. The main genera involved in bile acid metabolism include *Clostridium*, *Eubacterium*, and *Bacteroides* [60]. Secondary bile acids exert biological effects through the farnesoid X receptor (FXR) and G protein-coupled bile acid receptor 1 (TGR5/GPBAR1). FXR is highly expressed in the kidney [61], and TGR5 activation maintains mitochondrial homeostasis and attenuates renal injury via the SIRT1–PGC-1α axis [63].

UDCA is the most extensively studied protective bile acid. A breakthrough study found that the gut symbiont *Eubacterium limosum* can produce UDCA, which protects renal tubular epithelial cells from IRI by activating PPAR-γ to promote fatty acid oxidation and increase ATP production [23]. Clinical studies have confirmed that cardiac surgery patients with higher preoperative serum UDCA levels have significantly lower postoperative AKI incidence, and UDCA levels positively correlate with eGFR and negatively correlate with serum creatinine and NGAL [56]. UDCA has been used as a hepatoprotective drug for many years with a well-established safety profile. Additionally, bile acid accumulation can cause bile cast nephropathy [64]; in heavy metal-induced renal injury, UDCA reduces BUN, creatinine, and KIM-1 [24]. LCA and DCA regulate inflammation and fibrosis through FXR and GPBAR1 [25].

#### 3.1.3. Tryptophan Metabolites

Tryptophan metabolism is an important pathway of gut microbiota–host interaction. Microbiota metabolize tryptophan to produce indole and its derivatives, including indole-3-propionic acid (IPA) and indole-3-aldehyde (IAld). IPA, the main tryptophan metabolite of *Lactobacillus*, is an endogenous ligand of the aryl hydrocarbon receptor (AhR). IPA activates AhR signaling to upregulate intestinal barrier-related gene expression, maintain epithelial tight junction integrity, and reduce enterogenic inflammation and toxin translocation [65]. In AKI models, IPA attenuates tubular epithelial cell inflammatory responses and programmed cell death by inhibiting the NF-κB pathway and NLRP3 inflammasome activation [66]. IAld, also an AhR ligand, plays an important role in maintaining mucosal immune homeostasis and regulating IL-22 secretion [67]. Notably, some tryptophan metabolites (e.g., indole) can be converted to uremic toxins such as IS in the liver, suggesting that the tryptophan metabolic pathway has bidirectional protective–injurious properties, with the ultimate effect depending on the microbiota metabolic direction and host status [27,28].

#### 3.1.4. D-Amino Acids

D-amino acids are a recently discovered class of renoprotective metabolites. Gut microbiota can produce multiple D-amino acids, including D-alanine, D-serine, D-arginine, and D-aspartate, totaling 12 types [30]. Among them, D-alanine and D-serine have been confirmed to possess tubuloprotective effects [32]. D-amino acid synthetases and degradases exist in the kidney, participating in the metabolic regulation of D-amino acids [68].

In the HK-2 human proximal tubular cell hypoxia model, D-alanine hydrochloride and D-alanine dipeptide significantly improve cell survival, with mechanisms related to the regulation of cell cycle and cyclin B1 expression [29]. The effective concentration is 10 µM, and the combination of D-alanine with L-alanine is superior to either alone. D-serine promotes tubular cell proliferation and tissue remodeling via the mTOR signaling pathway [31]. Clinical studies have found that plasma D-serine levels correlate with the degree of renal function decline in AKI patients [69]. Oral D-serine attenuates kidney injury in both normal and D-serine knockout mice, inhibits hypoxia, and promotes tubular proliferation [32]. However, high-dose D-serine itself is nephrotoxic, suggesting that its protective effect is dose-dependent and bidirectional [70].

#### 3.1.5. Polyamines

Polyamines, including putrescine, spermine, and spermidine, are mainly produced by beneficial bacteria such as *Lactobacillus* and *Bifidobacterium*. Polyamines play important roles in maintaining intestinal epithelial integrity and barrier function by regulating cell proliferation, differentiation, and autophagy [26]. In AKI, the abundance of these polyamine-producing bacteria decreases, leading to reduced polyamine levels and impaired intestinal barrier repair. Studies have shown that exogenous spermine supplementation attenuates IRI-induced kidney injury, with mechanisms related to the inhibition of tubular epithelial cell apoptosis and regulation of autophagic flux [33]. Furthermore, polyamines can regulate macrophage polarization, promoting M2 macrophage differentiation and attenuating renal inflammation, playing an important role in the AKI recovery phase [35,36].

### 3.2. Toxic Metabolites

#### 3.2.1. Indoxyl Sulfate (IS)

IS is a protein-bound uremic toxin formed from tryptophan metabolized by gut microbiota (e.g., *Escherichia coli*) to indole, which is then conjugated by hepatic sulfotransferases. Serum IS levels are significantly elevated in AKI patients and closely correlate with kidney injury markers such as KIM-1 and NGAL [71]. IS is taken up by renal tubular epithelial cells via organic anion transporters (OATs), activates NADPH oxidase to induce reactive oxygen species (ROS) production, and causes oxidative stress and mitochondrial dysfunction [36]; downregulates eNOS expression and reduces NO production, leading to endothelial dysfunction [37]; activates the AhR/NF-κB pathway to promote TNF-α and IL-6 expression and macrophage infiltration [40]; and induces epithelial–mesenchymal transition (EMT) via the TGF-β/Smad pathway, accelerating AKI-to-CKD transition [72].

The oral adsorbent AST-120 can adsorb indole and other uremic toxin precursors in the gut, reducing IS generation and absorption. Animal studies have confirmed that AST-120 significantly reduces serum IS levels and attenuates IR- and cisplatin-induced AKI [73]. Due to the strong protein-binding capacity of IS, conventional dialysis has limited clearance efficiency, giving AST-120 a unique advantage in clearing enterogenic toxins. A breakthrough study demonstrated that knockout of the *Bacteroides* tryptophanase gene reduced IS production to below the detection limit, directly confirming the key role of microbiota tryptophan metabolism in IS generation [38]. Clinical studies have confirmed that elevated plasma IS levels are closely associated with mortality in hospital-acquired AKI patients [39], and IS levels positively correlate with RIFLE classification [74]. Furthermore, gestational IS exposure can increase blood–brain barrier permeability and is associated with pregnancy-related AKI (PR-AKI)-like injury [75].

#### 3.2.2. p-Cresyl Sulfate (PCS)

PCS is a protein-bound uremic toxin formed from tyrosine metabolized by gut microbiota to p-cresol, which is then conjugated by hepatic sulfotransferases. The nephrotoxic mechanisms of PCS are similar to but distinct from those of IS: PCS inhibits mitochondrial respiratory chain complexes, reducing ATP production and disrupting tubular epithelial cell energy metabolism [76]; downregulates eNOS expression, reducing NO production and causing renal vasoconstriction and local ischemia [77]; and disrupts endothelial cell–cell junctions, increasing vascular permeability and further impairing renal microcirculation [77]. Furthermore, IS and PCS exhibit synergistic toxicity, with combined exposure significantly worsening kidney injury. In AKI patients, elevated serum PCS levels are significantly associated with renal function deterioration, prolonged hospitalization, and adverse outcomes [41].

#### 3.2.3. Trimethylamine N-Oxide (TMAO)

TMAO is a metabolite formed when dietary choline, L-carnitine, and lecithin are metabolized by gut microbiota (e.g., *Clostridium*, *Escherichia*, *Desulfovibrio*) to trimethylamine (TMA) [42], which is then oxidized by hepatic flavin monooxygenases (FMOs). Clinical studies have shown that serum TMAO levels are significantly elevated in AKI patients, correlating with renal function decline, inflammatory responses, and adverse outcomes, and elevated TMAO levels increase the risk of cardiovascular disease and renal failure [78]. In cardiac surgery patients, preoperative TMAO levels positively correlate with postoperative AKI incidence [42].

The nephrotoxic mechanisms of TMAO involve multiple pathways. TMAO activates the NLRP3 inflammasome, promoting ROS production and NO reduction, leading to microcirculatory dysfunction [79]. TMAO promotes macrophage M1 polarization via the NF-κB pathway, releasing pro-inflammatory cytokines including TNF-α, IL-6, and IL-1β [43]. Notably, the CCR2+ macrophage subset is the main responder to TMAO; TMAO promotes the interaction between this subset and tubular cells, exacerbating kidney injury. *CCR2* gene knockout or the CCR2 antagonist RS-102895 significantly attenuates inflammation and fibrosis [44]. TMAO induces ROS production via the NADPH oxidase pathway, promoting tubular cell apoptosis [79]. Furthermore, TMAO promotes renal interstitial fibroblast activation and extracellular matrix deposition via the TGF-β/Smad signaling pathway [45], and long-term TMAO exposure can lead to CKD, heart failure, and fatty liver fibrosis [80]. Animal studies have confirmed that a high-choline diet elevates TMAO levels and worsens IR-induced AKI and CKD progression [81]. DHA-acylated curcumin diester can attenuate post-AKI cardiovascular injury by inhibiting the TMAO-mediated PI3K/AKT/NF-κB signaling pathway [82].

#### 3.2.4. Endotoxin (LPS)

LPS is a major component of the Gram-negative bacterial cell wall that translocates into the bloodstream when gut dysbiosis and barrier impairment occur. LPS activates renal intrinsic cells and infiltrating immune cells via the Toll-like receptor 4 (TLR4)/myeloid differentiation factor 88 (MyD88)/NF-κB signaling pathway, inducing the expression of large quantities of pro-inflammatory cytokines and triggering renal inflammation [46]. In sepsis-associated AKI, LPS-mediated systemic inflammation leads to hemodynamic disturbances, forming a “systemic hypotension–renal local vasoconstriction” perfusion pattern that aggravates renal ischemic injury [83]. Furthermore, LPS can activate the complement system, promote neutrophil extracellular trap (NET) formation and microvascular thrombosis, further amplifying inflammatory injury. Clinical studies have confirmed that elevated circulating LPS levels in AKI patients are closely associated with renal function deterioration, multiple organ dysfunction, and increased mortality [47].

## 4. Key Mechanisms of Gut Microbiota Metabolites Driving AKI

The preceding sections have described the direct effects of individual metabolites on the kidney. However, in the actual pathological process of AKI, multiple metabolites do not act in isolation but form a synergistic injury network through common downstream pathways (oxidative stress, inflammation activation, endothelial dysfunction, and hemodynamic disturbance), constituting a bidirectional feedback loop of the “gut–kidney axis” with the intestinal barrier, immune system, and circulatory system. This chapter focuses on the core mechanistic nodes of this network from an integrative perspective, aiming to reveal the overall pathophysiological logic of microbiota metabolites driving AKI.

### 4.1. Bidirectional Interaction and Vicious Cycle of the Gut–Kidney Axis

The core feature of the gut–kidney axis is the bidirectional feedback regulation between the gut and kidney: gut dysbiosis can aggravate kidney injury, while AKI itself can further worsen gut microbiota dysbiosis, forming a self-sustaining vicious cycle. In the “gut-to-kidney” direction, intestinal barrier impairment in AKI allows continuous translocation of toxic metabolites such as IS, PCS, TMAO, and LPS into the bloodstream, which reach the kidney via the circulation and damage tubular epithelial cells and renal interstitium through common pathways including oxidative stress, inflammation activation, and endothelial dysfunction (detailed in Section 3), aggravating renal structural and functional damage [73]. In the “kidney-to-gut” direction, accumulation of metabolic wastes such as serum creatinine and BUN in AKI alters the intestinal environment (pH, osmolarity, oxygen partial pressure), selectively inhibiting beneficial bacteria and promoting opportunistic pathogen proliferation [84]. Meanwhile, systemic inflammation and oxidative stress accompanying AKI downregulate the expression of tight junction proteins including ZO-1, occludin, and claudin-1, further disrupting the intestinal barrier and exacerbating toxin translocation [38].

The clinical significance of this bidirectional interaction lies in the fact that kidney-targeted therapy alone, without concurrent correction of gut dysbiosis, often fails to interrupt the vicious cycle [85]. Conversely, gut-targeted interventions can indirectly improve renal prognosis by reducing enterogenic toxin load and restoring microbiota metabolic balance. Recent studies suggest that gut microbiota in AKI may influence sympathetic nerve activity and the hypothalamic–pituitary–adrenal axis via the “gut–brain–kidney” axis, indirectly regulating renal hemodynamics and inflammatory responses (this mechanism remains to be confirmed) [86]. Understanding the molecular nodes of this vicious cycle is a theoretical prerequisite for developing effective intervention strategies.

**Figure 1:**
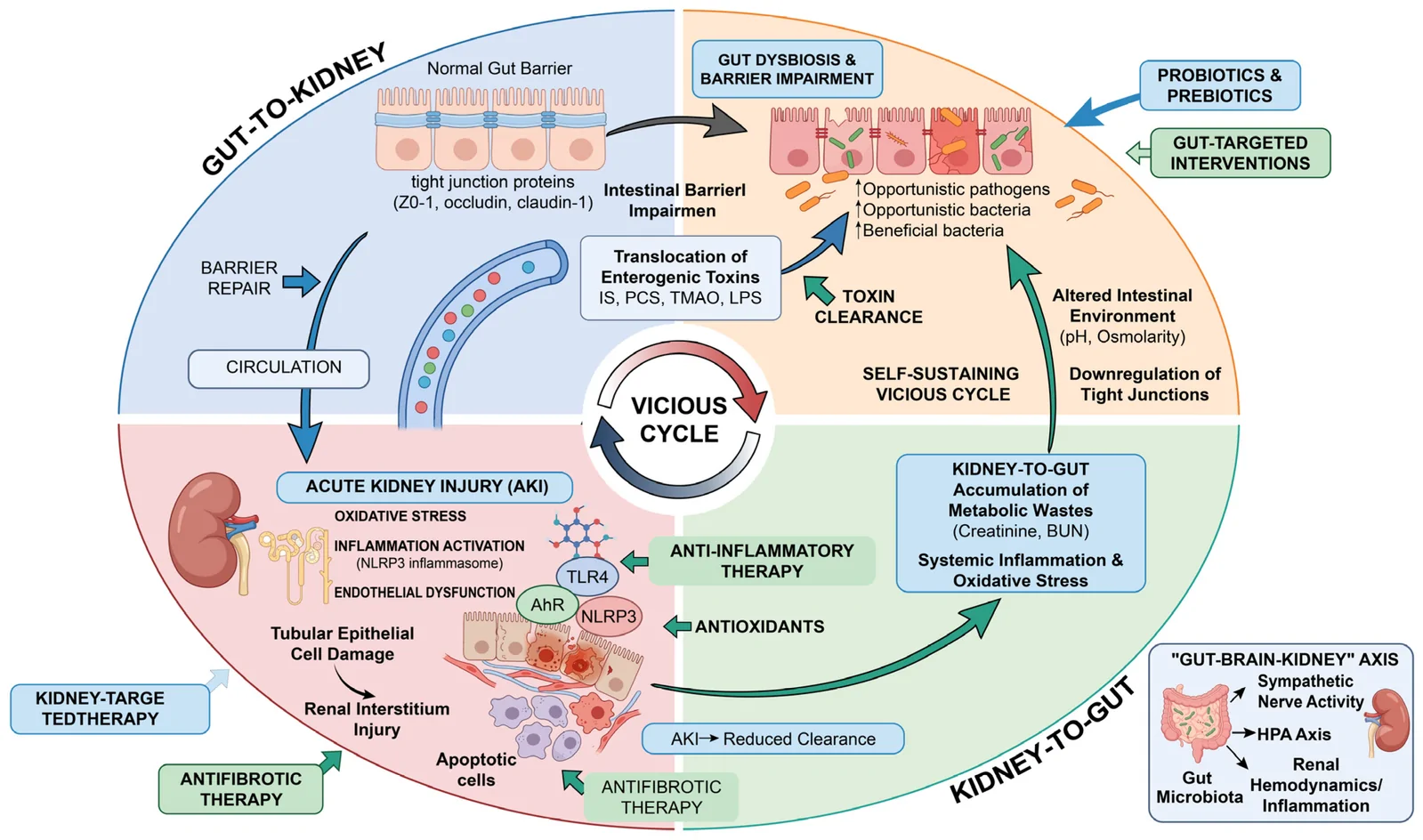
Schematic diagram of the AKI gut–kidney axis vicious cycle.

From a temporal dynamics perspective, the gut–kidney axis vicious cycle exhibits differential characteristics between the acute phase and the chronic transition phase. In the acute phase of AKI, intestinal barrier disruption and toxin translocation are the dominant events, with enterogenic toxins rapidly entering the bloodstream and aggravating acute tubular injury. In the chronic transition phase, persistent low-grade inflammation and microbiota metabolic dysregulation drive the AKI-to-CKD transition, with toxins such as IS and TMAO promoting renal interstitial fibrosis via the TGF-β/Smad pathway, leading to irreversible renal structural remodeling. This temporal difference suggests that acute-phase interventions should focus on barrier repair and toxin clearance, while chronic transition-phase interventions need to address both microbiota metabolic remodeling and antifibrotic therapy (Figure 1).

### 4.2. Remodeling of the Immune Cell Network

Immune–inflammatory imbalance is a core pathological feature of AKI renal tissue injury, and gut microbiota and their metabolites are key upstream factors shaping the renal immune cell network. Upon AKI onset, the kidney exhibits a pro-inflammatory-dominant immune remodeling: pro-inflammatory cell populations including M1 macrophages, Th17 cells, and neutrophils expand and release pro-inflammatory cytokines such as TNF-α, IFN-γ, IL-6, and IL-17, driving tubular epithelial cell injury and interstitial inflammation. Meanwhile, anti-inflammatory/repair cell populations including M2 macrophages and Treg cells are relatively insufficient, leading to impaired inflammation resolution and tissue repair [39]. This immune homeostatic imbalance directly participates in the pathological process of renal tissue injury.

Gut microbiota remotely regulate renal immune cell differentiation and function through their metabolites. Jang et al. demonstrated that Th17 and Th1 cell proportions are significantly elevated in the kidneys of germ-free mice, and this immune activation state is reversed after fecal microbiota transplantation from normal-flora mice [74], directly proving the role of microbiota in shaping renal immune homeostasis. Mechanistically, protective metabolites (e.g., SCFAs promoting Treg differentiation via HDAC inhibition, IPA maintaining mucosal immune homeostasis via AhR signaling) and toxic metabolites (e.g., TMAO promoting M1 polarization via NLRP3 activation, IS amplifying inflammation via AhR/NF-κB) form a dynamic “anti-inflammatory versus pro-inflammatory” balance in the AKI kidney [87]. When dysbiosis leads to reduced protective metabolites and increased toxic metabolites, the balance shifts toward a pro-inflammatory direction, driving kidney injury progression. Furthermore, microbiota metabolites can indirectly influence systemic immune status by regulating intestinal mucosal immunity (e.g., IL-22 secretion, IgA production), further affecting renal inflammatory responses.

From an organ crosstalk perspective, the immune regulation of the gut–kidney axis is embedded in a broader “gut–liver–kidney” immune network. The liver, as the first-pass metabolic organ for enterogenic toxins, directly influences the toxin load reaching the kidney. Meanwhile, hepatic Kupffer cell activation releases systemic pro-inflammatory mediators that synergize with renal intrinsic immune cells. This multi-organ immune crosstalk suggests that future intervention strategies may need to simultaneously target the gut, liver, and kidney to effectively block systemic inflammatory cascades [88].

### 4.3. Integrative Effects of Hemodynamic Disturbance

**Figure 2:**
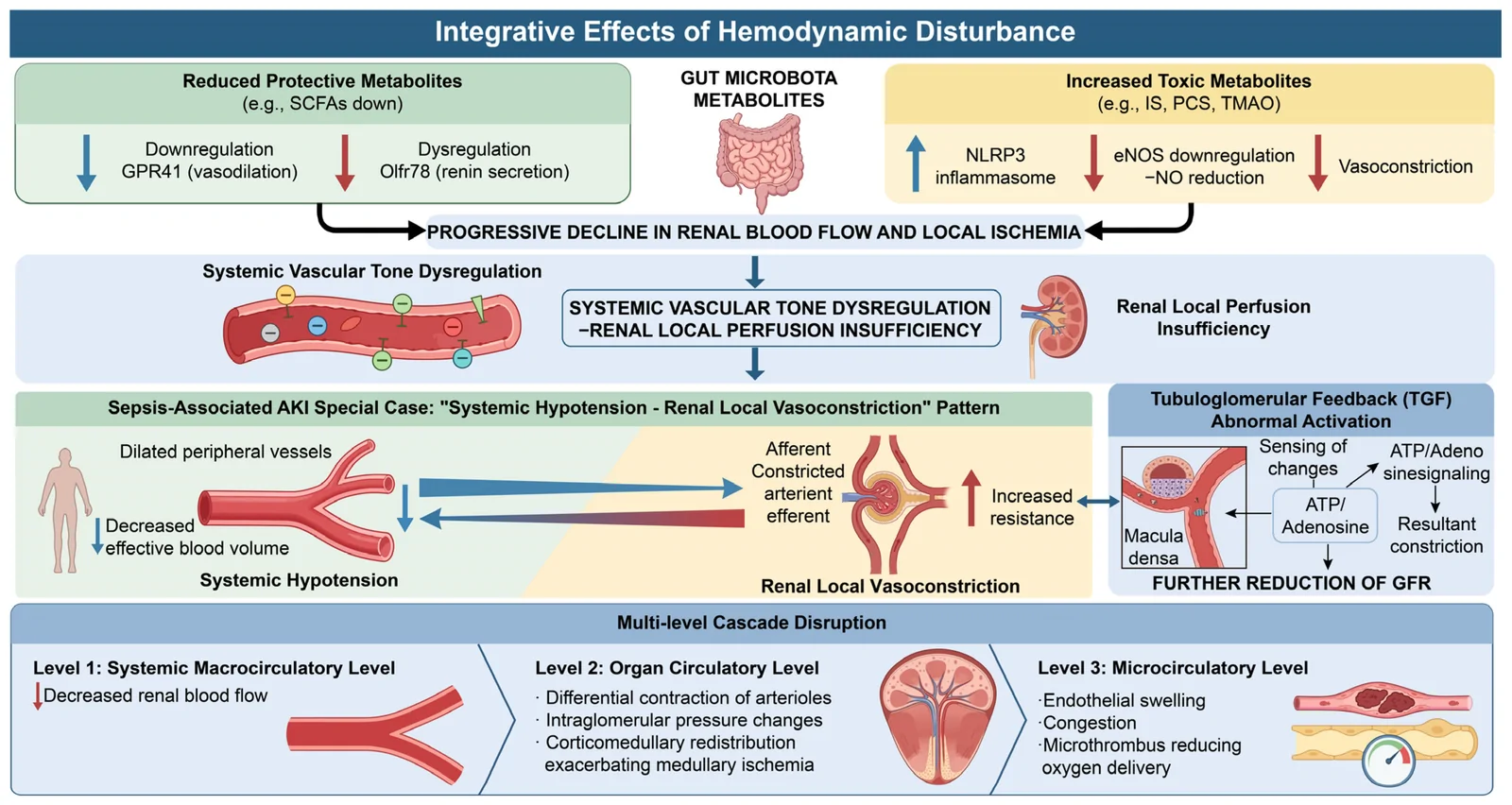
Schematic diagram of the AKI hemodynamic disturbance cascade.

Hemodynamic disturbance is a core pathological link between gut microbiota metabolites and AKI. Multiple protective and toxic metabolites converge on vascular regulation, renal perfusion, and microcirculatory dysfunction, forming a hierarchical hemodynamic disturbance cascade (Figure 2). During AKI, multiple metabolites synergistically disrupt renal microcirculatory homeostasis, forming an integrative hemodynamic disturbance characterized by “systemic vascular tone dysregulation–renal local perfusion insufficiency.” On one hand, the reduction in protective metabolites such as SCFAs weakens their regulatory effects on vasodilation via GPR41 and renin secretion via Olfr78, leading to imbalanced renal vascular tone regulation. On the other hand, toxic metabolites such as IS, PCS, and TMAO cause renal vasoconstriction and microcirculatory dysfunction through the aforementioned NLRP3 inflammasome activation and eNOS/NO downregulation mechanisms (detailed in Section 3.2). The net effect of the imbalance between these two metabolite categories is progressive decline in renal blood flow and exacerbation of renal local ischemia.

In sepsis-associated AKI, hemodynamic disturbance exhibits a unique “systemic hypotension–renal local vasoconstriction” perfusion pattern (detailed in Section 3.2.4): LPS-mediated systemic inflammation leads to peripheral vasodilation and decreased effective circulating blood volume, while reactive vasoconstriction of renal vessels in response to inflammatory mediators and toxic metabolites increases renal vascular resistance, leading to further deterioration of renal perfusion. Furthermore, tubuloglomerular feedback (TGF) is abnormally activated in AKI, with the macula densa sensing changes in tubular fluid flow and contracting afferent arterioles via the ATP/adenosine signaling pathway, further reducing glomerular filtration rate. This integrative “systemic–renal local” hemodynamic disturbance is an important pathological basis for the high mortality of sepsis AKI and suggests that microbiota metabolite-targeted interventions may exert renoprotective effects by improving hemodynamics.

From a microcirculation–macrocirculation integrative perspective, AKI hemodynamic disturbance exhibits multi-level characteristics: at the macrocirculatory level, systemic vascular tone dysregulation leads to decreased renal blood flow; at the organ circulation level, differential contraction/dilation of afferent and efferent arterioles alters intraglomerular pressure, and abnormal corticomedullary blood flow distribution exacerbates outer medullary ischemia; at the microcirculatory level, endothelial swelling, peritubular capillary congestion, and microthrombus formation further reduce oxygen delivery. This three-level cascade disruption makes actual renal tissue oxygen supply far below the level suggested by hemodynamic parameters, and microbiota metabolite-targeted interventions hold promise for simultaneously improving multiple levels of disturbance from the source.

## 5. Gut Microbiota as Therapeutic Targets for AKI

The gut–kidney axis theory has highlighted gut microbiota and their metabolites as promising therapeutic targets for AKI prevention and treatment (Table 2). Current intervention strategies can be broadly categorized according to their primary targets into two approaches: one targets the gut microbiota itself and aims to restore metabolic balance by modulating its composition and structure through probiotics/synbiotics, prebiotics/dietary interventions, and fecal microbiota transplantation; the other targets gut microbiota metabolites directly, regulating metabolite levels by supplementing protective metabolites or clearing toxic metabolites. The two categories have distinct characteristics in terms of mechanisms and clinical translation maturity, and they complement each other.

### 5.1. Therapies Targeting Gut Microbiota

#### 5.1.1. Probiotics and Synbiotics

Probiotics exert renoprotective effects by colonizing the gut, competitively inhibiting pathogen growth, repairing the intestinal barrier, and regulating immune responses. In the IRI-induced AKI mouse model, *Lactobacillus salivarius* pretreatment significantly reduces serum creatinine and BUN levels, attenuates tubular necrosis and cell apoptosis, with mechanisms related to downregulation of the TLR4/NF-κB pathway and inhibition of inflammatory cytokine expression [41]. Synbiotics, combining probiotics and prebiotics, synergistically promote beneficial bacteria colonization and metabolic activity. In cisplatin-induced AKI, a synbiotic preparation containing *Bifidobacterium* and fructooligosaccharides significantly attenuates kidney injury while increasing SCFA-producing bacteria abundance.

The renoprotective effects of different probiotic strains exhibit significant strain specificity. In addition to *L. salivarius*, *Bifidobacterium bifidum* BGN4 and multi-strain microbial cocktail preparations have been confirmed to attenuate AKI-associated inflammatory responses and kidney injury [89]. *Lactobacillus casei* Zhang slows the progression of acute and chronic kidney disease by alleviating intestinal inflammation, repairing mucosal damage, and enhancing colonic barrier protein occludin expression [90]. In the LPS-induced AKI mouse model, *Akkermansia muciniphila* pretreatment significantly reduces serum creatinine and BUN levels, attenuates tubular necrosis and apoptosis, and increases the abundance of beneficial bacteria such as *Lactobacillus*, altering metabolic pathways including tyrosine metabolism and amino acid homeostasis [53]. Clinical studies of *Lactobacillus acidophilus* KBL409 have shown that 16 weeks of continuous KBL409 administration significantly reduces serum IS concentrations [91].

#### 5.1.2. Prebiotics and Dietary Interventions

Prebiotics are dietary components that selectively stimulate the growth and activity of beneficial bacteria, mainly including dietary fibers and oligosaccharides. As the main substrate for SCFA production, dietary fiber promotes the generation of acetate, propionate, and butyrate by increasing the abundance of SCFA-producing bacteria, exerting anti-inflammatory and renoprotective effects. Caloric restriction, as a dietary intervention strategy, has shown unique value in AKI protection. Studies have confirmed that caloric restriction enriches the gut symbiont *Parabacteroides goldsteinii* (PG), which attenuates IR-induced kidney injury by enhancing intestinal barrier function and upregulating tight junction protein expression. Notably, live bacterial colonization, rather than heat-killed bacteria, is required for the protective effect, suggesting dependence on microbial metabolic activity [92]. Overall, evidence for prebiotic and dietary interventions in AKI specifically remains limited, resting largely on this single caloric-restriction/*Parabacteroides* study and related dietary-fiber-SCFA data described above (Section 3.1.1); broader dietary intervention trials in AKI populations are lacking, and this should be regarded as an emerging rather than established therapeutic avenue.

#### 5.1.3. Fecal Microbiota Transplantation

Fecal microbiota transplantation (FMT) restores microbial community homeostasis by transferring gut microbiota from healthy donors to recipients via capsules or colonoscopy [78]. FMT can reduce the levels of protein-bound uremic toxins including IS, PCS, TMAO, and phenylacetylglycine, thereby attenuating kidney injury [42]. In the IRI rat model, FMT exerts renoprotective effects by restructuring the microbiota, enriching SCFA-producing bacteria such as Lachnospiraceae, and increasing propionate production [79]. Mechanistic studies have further confirmed that the protective effect of FMT is closely related to restoring microbiota homeostasis and enhancing barrier function: germ-free mice receiving microbiota transplantation from IRI mice show elevated TNF-α and IFN-γ mRNA levels in kidney tissue [43], and FMT reverses this immune activation state by reestablishing a healthy microbiota structure. Global clinical trial analysis shows that FMT accounts for 15.2% of 46 gut microbiota-related kidney disease clinical trials [44]. A prospective study showed that repeated FMT in CKD patients reduced 24 h urinary protein [45]. However, the standardization of this method remains incomplete, and key parameters including delivery route, dose, and timing require further clarification.

#### 5.1.4. Safety Considerations for Microbiota-Targeted Interventions

Microbiota-targeted interventions are not risk-free, particularly in critically ill AKI patients with impaired intestinal barrier function. Live probiotic organisms and FMT both carry a recognized, if uncommon, risk of bacterial translocation and secondary bloodstream infection or sepsis, especially in immunocompromised, critically ill, or barrier-disrupted hosts; probiotic-associated bacteremia and fungemia have been reported in vulnerable populations in the wider critical-care literature. Indeed, whole-genome sequencing has directly demonstrated transmission of probiotic-derived *Lactobacillus* from administered capsules to the bloodstream of intensive care unit patients, confirming that translocation of live organisms is a genuine rather than theoretical hazard in this setting [93]. FMT additionally carries donor-screening and transmissible-pathogen risks—illustrated by the documented transmission of extended-spectrum β-lactamase-producing *Escherichia coli* from an inadequately screened donor, which caused bacteremia in two recipients and proved fatal in one [94]—and standardized safety monitoring protocols for AKI populations are not yet established. These risks likely vary with the severity of gut barrier disruption and the degree of immunosuppression, and should be weighed against the anticipated renoprotective benefit on a patient-by-patient basis; they are not systematically addressed in most of the animal studies summarized above and warrant explicit safety endpoints in future clinical trials.

### 5.2. Therapies Targeting Gut Metabolites

#### 5.2.1. Supplementation of Protective Metabolites

SCFAs, UDCA, and D-serine have shown renoprotective effects in preclinical studies and, for some compounds, preliminary clinical investigations, suggesting their potential as therapeutic strategies for AKI. As described above (Section 3.1.1), SCFAs exert immunomodulatory and barrier-protective effects through GPR41/43/109a activation and HDAC inhibition; increasing dietary fiber intake or direct SCFA supplementation is an important approach to elevating protective metabolite levels [95]. High-fiber diets can effectively delay the AKI-to-CKD transition: in the folic acid nephropathy mouse model, a high-fiber diet attenuated tubular injury on day 2 and significantly improved interstitial fibrosis and chronic inflammation on day 28 [95]. However, the short half-life and poor targeting of SCFAs remain major obstacles to clinical translation.

Among the protective metabolites discussed, UDCA currently has relatively more human observational evidence. As described above (Section 3.1.2), UDCA exerts renoprotective effects by activating the TGR5 receptor and inhibiting endoplasmic reticulum stress and oxidative stress. Given its long-standing clinical use in liver disease and well-established safety profile, UDCA may represent a promising candidate for further clinical evaluation in AKI. Furthermore, the renoprotective effects of D-amino acids have entered preliminary clinical investigation: the latest multicenter randomized controlled trial showed that healthy volunteers receiving oral D-alanine at 6 g/day for 7 days had elevated eGFR with good tolerability [96]. Due to the dose-dependent nature of D-alanine protective effects, strict dose control is required [70]. The clinical value of D-amino acids in AKI patients awaits validation in large-scale clinical trials.

#### 5.2.2. Clearance of Toxic Metabolites

The oral adsorbent AST-120 can adsorb indole and other uremic toxin precursors in the gut, reducing IS generation and absorption. Animal studies have confirmed that AST-120 treatment significantly reduces serum IS levels and attenuates IR- and cisplatin-induced AKI [46,47]. Due to the strong protein-binding capacity of IS, conventional dialysis has limited clearance efficiency, giving AST-120 a unique advantage in clearing enterogenic toxins. Hemodialysis and hemoperfusion can clear some toxic metabolites from the circulation: TMAO, with its small molecular weight and water solubility, can be cleared by dialysis; IS and PCS, with their strong protein-binding capacity, require high-flux dialysis or combined hemoperfusion. In recent years, the development of novel adsorbent materials and targeted microbiota enzyme inhibitors (e.g., TMA lyase inhibitors) has provided new directions for precision clearance of toxic metabolites, although most remain in preclinical research stages.

## 6. Summary and Perspectives

The dysbiosis of gut microbiota and their metabolites and AKI mutually influence each other, constituting a vicious cycle of bidirectional feedback in the “gut–kidney axis.” The increase in uremic toxins (IS, PCS, TMAO, LPS) and the insufficiency of protective metabolites (SCFAs, UDCA, D-amino acids, polyamines) are key drivers of AKI progression, and the imbalance between the two aggravates kidney injury through common pathways including oxidative stress, inflammation activation, immune remodeling, and hemodynamic disturbance. Gut microbiota-based interventions have shown potential to improve prognosis in animal models and preliminary clinical studies, but still face numerous challenges.

### Limitations

This review has several limitations. First, as a narrative rather than a systematic review, article selection was not governed by a pre-registered protocol and is subject to selection bias; effect estimates and evidence-level judgments described here should be interpreted qualitatively rather than as pooled or validated quantitative conclusions. Second, the great majority of the mechanistic evidence linking specific microbiota metabolites to AKI derives from animal models and in vitro systems; human data are largely observational, and interventional randomized controlled trials in AKI patients remain scarce for nearly all strategies discussed, including for UDCA and D-amino acid supplementation. Third, in the available human studies, numerous potential confounders—including sepsis, antibiotic exposure, critical illness, hospitalization, dietary changes, concomitant medications, hemodynamic instability, and pre-existing chronic kidney disease—can independently alter gut microbiota composition and intestinal permeability, making it difficult to establish the directionality of the gut–kidney relationship from observational data alone; this should be borne in mind when interpreting the clinical associations summarized throughout this review. Fourth, the classification of metabolites as strictly “protective” or “toxic” (Table 1) is a simplification of effects that are often dose-, context-, tissue-, and disease-stage-dependent. Finally, heterogeneity in microbiota profiling methods (16S rRNA sequencing versus shotgun metagenomics), AKI definitions, and outcome measures across the cited studies limits direct comparability between them.

Future research should focus on the following directions: first, to elucidate the molecular mechanisms of gut microbiota–AKI interaction, defining the details of microbiota–metabolite–receptor–renal target cell signaling pathways to provide targets for precision intervention; second, to conduct large-scale, multicenter, randomized controlled clinical trials to validate the efficacy and safety of microbiota modulation strategies in AKI patients and establish personalized treatment protocols based on microbiota characteristics; third, to promote the clinical translation of metabolite-targeted therapeutic strategies, with UDCA and D-amino acids representing promising candidates for further clinical evaluation; and fourth, to integrate multi-omics technologies (metagenomics, metabolomics, single-cell sequencing) to construct a precision molecular classification system for the AKI gut–kidney axis, providing novel insights for early recognition and personalized treatment of AKI.

## Figures and Tables

**Table 1:** Classification of gut microbiota metabolites in AKI.

Category	Representative Metabolite	Main Source Microbiota	Core Mechanism	Key References
Protective	SCFAs (acetate/propionate/butyrate)	*Faecalibacterium*, *Roseburia*, *Ruminococcus*	GPR41/43/109a activation, HDAC inhibition	[17,19,20,21]
Protective	Secondary bile acids (UDCA, LCA)	*Bacteroides*, *Bifidobacterium*	TGR5/FXR signaling, mitochondrial protection	[22,23,24,25]
Protective	Tryptophan metabolites (IPA, IAld)	*Lactobacillus*, *Clostridium*	AhR activation, aryl hydrocarbon receptor pathway	[26,27,28]
Protective	D-amino acids (D-Ser, D-Ala)	Various microbiota	Competitive antagonism of nephrotoxicity	[29,30,31,32]
Protective	Polyamines (putrescine, spermine)	*Lactobacillus*, *Bifidobacterium*	Autophagy regulation, barrier maintenance	[33,34,35]
Toxic	Indoxyl sulfate (IS)	*Clostridium*, *Escherichia coli*	AhR/NADPH oxidase/ROS	[36,37,38,39]
Toxic	p-Cresyl sulfate (PCS)	*Clostridium*, *Bacteroides*	eNOS inhibition, mitochondrial damage	[40,41]
Toxic	Trimethylamine N-oxide (TMAO)	*Clostridium*, *Escherichia coli*	NLRP3 inflammasome, ER stress	[42,43,44,45]
Toxic	Endotoxin (LPS)	Gram-negative bacteria	TLR4/MyD88/NF-κB pathway	[46,47]

This table summarizes the source microbiota, representative metabolites, and core mechanistic targets of each metabolite class, with key supporting references listed for each row. Abbreviations: SCFAs, short-chain fatty acids; UDCA, ursodeoxycholic acid; LCA, lithocholic acid; IPA, indole-3-propionic acid; IAld, indole-3-aldehyde; D-Ser, D-serine; D-Ala, D-alanine; AhR, aryl hydrocarbon receptor; GPR, G-protein-coupled receptor; HDAC, histone deacetylase; TGR5/FXR, G protein-coupled bile acid receptor 1/farnesoid X receptor; IS, indoxyl sulfate; PCS, p-cresyl sulfate; TMAO, trimethylamine N-oxide; LPS, lipopolysaccharide. The “Protective” and “Toxic” category labels reflect the predominant direction of effect reported in the cited literature; several of these metabolites show context-, dose-, or tissue-dependent effects (see Section 3 for details).

**Table 2:** Summary of gut microbiota-based intervention strategies for AKI.

Strategy Type	Representative Intervention	Mechanism	Evidence Type	Main Limitations
Microbiota-targeted	Probiotics (*Lactobacillus*, *Bifidobacterium*)	Competitive pathogen inhibition, barrier repair, immune regulation	Animal + limited clinical	Strain specificity, inconsistent dosing
Microbiota-targeted	Synbiotics (probiotics + prebiotics)	Synergistic promotion of beneficial bacteria colonization	Animal + limited clinical	Combination optimization needed
Microbiota-targeted	Prebiotics/dietary fiber	Increased SCFA-producing bacteria abundance	Animal + limited clinical	Large individual variation
Microbiota-targeted	Caloric restriction	Enrichment of *Parabacteroides* and other symbionts	Animal	Poor clinical compliance
Microbiota-targeted	Fecal microbiota transplantation (FMT)	Holistic microbiota restructuring	Animal + limited clinical	Insufficient standardization, safety
Metabolite-targeted	SCFA/high-fiber diet supplementation	Direct supplementation of protective metabolites	Animal + limited clinical	Pharmacokinetics unclear
Metabolite-targeted	UDCA supplementation	TGR5 activation, ER stress inhibition	Observational clinical evidence (single cohort); mechanistic/animal evidence stronger than clinical	Requires RCT validation in AKI patients; indications to expand
Metabolite-targeted	D-amino acid supplementation	Competitive antagonism of nephrotoxicity	Phase I clinical	Narrow dose window
Metabolite-targeted	AST-120 oral adsorbent	IS precursor adsorption, reduced absorption	Animal + limited clinical	Long-term compliance
Metabolite-targeted	High-flux dialysis/hemoperfusion	Clearance of circulating toxic metabolites	Clinical application	Limited for protein-bound toxins

This table summarizes representative strategies and the types of supporting evidence currently available. Most interventions remain predominantly supported by animal-model evidence, with human data limited to small or observational studies unless otherwise noted. Interventional randomized controlled trials in AKI patients remain largely lacking; therefore, the “Evidence Type” designations describe the nature of the available evidence rather than its strength.

## Data Availability

No new data were created or analyzed in this study. Data sharing is not applicable to this article.

## Abbreviations

The following abbreviations are used in this manuscript:

AKI	Acute Kidney Injury
CKD	Chronic Kidney Disease
SCFAs	Short-Chain Fatty Acids
IS	Indoxyl Sulfate
PCS	p-Cresyl Sulfate
TMAO	Trimethylamine N-Oxide
LPS	Lipopolysaccharide
IRI	Ischemia–Reperfusion Injury
FMT	Fecal Microbiota Transplantation
UDCA	Ursodeoxycholic Acid
